# Long non-coding RNA NCK1-AS1 is overexpressed in esophageal squamous cell carcinoma and predicts survival

**DOI:** 10.1080/21655979.2022.2038449

**Published:** 2022-03-21

**Authors:** Xin Fu, Xi Chen, Yuanyuan Si, Youjie Yao, Zhengming Jiang, Kui Chen

**Affiliations:** Department of Cardiology, The First Affiliated Hospital of Zhengzhou University, Zhengzhou City, P. R. China

**Keywords:** Esophageal squamous cell carcinoma, lncRNANCK1-AS1, TGF-β1, survival, regulation

## Abstract

Long noncoding RNAs have shown pivotal regulatory roles in tumorigenesis and progression. NCK1-AS1 promotes cervical cancer, while its involvement in esophageal cancer is hardly known. This study enrolled 52 esophageal squamous cell carcinoma (ESCC) patients (30 males and 22 females) at the average age of 56.4 ± 6.6 years in the range from 46 to 70 years, explored the involvement of NCK1-AS1 in ESCC, and analyzed the possible interaction between NCK1-AS1 and TGF-β signaling. Changes in gene expression were analyzed using RT-qPCR and Western blot. Interactions between gene expressions were analyzed using ESCC cells with transient transfections. Cell invasion and migration were analyzed using Transwell assays. Our data showed that plasma NCK1-AS1 was overexpressed in ESCC patients and positively correlated with NCK1-AS1 expression in tumor tissues but not in non-tumor tissues. Moreover, high plasma NCK1-AS1 levels were accompanied with poor survival. TGF-β1 expression level was also increased in tumor tissues compared to the adjacent normal tissues and positively correlated with NCK1-AS1 in tumor tissues. TGF-β1 overexpression in ESCC cells did not affect NCK1-AS1 expression, while NCK1-AS1 overexpression in ESCC cells upregulated TGF-β1. Moreover, TGF-β1 and NCK1-AS1 overexpression increased ESCC cell migration and invasion, while TGF-β inhibitor reduced the effects of NCK1-AS1 overexpression. Overall, NCK1-AS1 may promote ESCC by upregulating TGF-β1.

## Graphical abstract

**Figure:**
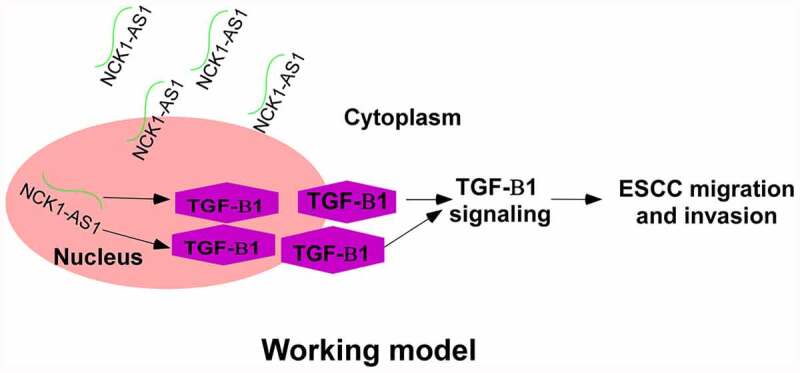

## Introduction

The incidence of esophageal cancer ranks in the 8^th^ place among all malignancies [1]. Due to its extreme malignant nature, esophageal cancer is also the 6^th^ leading cause of death among all cancer patients [1]. Esophageal squamous cell carcinoma (ESCC) is one of the major two esophageal cancer subtypes based on the histological findings [2]. ESCC accounts for more than 90% of esophageal cancer in Asian countries, such as China [3]. ESCC is now considered a major burden of public health in China. More than 50% of newly diagnosed ESCC are in China [4]. Early diagnosis of ESCC is difficult due to the lack of classic symptoms. Therefore, most ESCC patients are diagnosed with regional lymph node metastasis, local invasion, or even distant invasion by the time of initial diagnosis [5], leading to poor prognosis [6].

**Table 1.** Correlation between NCK1-AS1 expression and clinical characteristics of patients with ESCC

Clinical parameters	Low expression (N = 28)	High expression (N = 24)	P value
Age (years) <60 >60	14 14	10 14	P > 0.05
Gender Male Female	17 11	13 11	P > 0.05
Differentiation grade Well/moderate Poor	20 8	19 5	P < 0.05
TNM stage I/II III/IV	19 9	9 15	P < 0.01
Lymph node metastasis Negative Positive	20 8	14 10	P < 0.01

Although environmental factors affect ESCC, it is generally believed that genetic factors are major players in ESCC [7]. Long (>200 nt) non-coding RNAs (lncRNAs) are RNA transcripts without protein-coding ability but have important functions in cancer biology by regulating their downstream oncogene or tumor suppressors [8,9] and cancer-related signaling pathways. For example, lncMALAT1 is regulated by TGF-β1, an important player in tumor initiation and development, to promote ESCC invasion by inducing EMT [10]. LncRNA RMRP might serve as a tumor promoter to accelerate cell proliferation, migration, and invasion of ESCC through regulating the miR-613/NRP2 axis. LncRNA DDX11-AS1 regulates SNAI1/ZEB2 expression and activates the Wnt/β-catenin pathway via sponging miR-30d-5p as an epithelial-mesenchymal transition (EMT)-related lncRNA to advance ESCC progression, indicating it might serve as a therapeutic target for ESCC [11]. It has been reported that NCK1-AS1 is overexpressed in cervical cancer and promotes cervical cancer development. NCK1-AS1 inhibition suppressing cell proliferation and migration via reducing miR-134 expression [12]. Song et al. also showed that NCK1-AS1/miR-6857/CDK1 crosstalk serves as a critical effector in cervical cancer progression. Zhang et al. believed that NCK1-AS1 might elevate TRIM24 expression to further activate Wnt/β-catenin pathway via acting as a ceRNA for miR-138-2-3p. NCK1-AS1 silencing inhibits glioma progression [13]. However, its involvement in esophageal cancer is barely known. Therefore, we hypothesized that NCK1-AS1 expression is dysregulated in ESCC and this dysregulation carries important clinical significance. To test our hypothesis, we determined NCK1-AS1 level, evaluated its prognostic value for ESCC patients, explored its relationship with TGF-β1, and investigated their functions in ESCC development.

## Methods

### Research subjects

From May 2009 to May 2013, the First Affiliated Hospital of Zhengzhou University admitted 98 patients with ESCC. Among these patients, 52 cases (30 males and 22 females, 46 to 70 years, 56.4 ± 6.6 years) were enrolled in this study. The inclusion criteria were 1) no therapies received before admission and 2) willing to join the 5-year follow-up (5-year). The exclusion criteria were 1) had other medical conditions, 2) received treatment before admission, and 3) had a history of previous malignancy. Patients were classified into stage I (n = 12), II (n = 16), III (n = 14), and IV (n = 10) based on the staging criteria proposed by AJCC. Different treatments, such as esophagectomy, radiotherapies, chemotherapies, and their combinations were performed. All patients signed informed consent before admission. This study was approved by the Ethics Committee of the First Affiliated Hospital of Zhengzhou University.

### Specimen collection and cell lines

ESCC and paired non-tumor tissues were collected by fine-needle aspiration from all patients prior to therapies and stored at −80°C. Prior to therapy, blood (5 ml) samples were collected from 12 h-fastened patients in EDTA tubes and centrifuged at 1200 g for 15 min to collect plasm samples. EC109 and KYSE150 cell lines were purchased from ATCC (USA) and cultured in RPMI-1640 medium supplemented with 10% FBS at 37°C in an incubator with 5% CO_2_. all experiments were performed with mycoplasma-free cells.

### Follow-up

Patients were monitored by follow-up monthly via phone calls for 5 years, and deaths caused by factors unrelated to ESCC were excluded.

### Luciferase reporter assay

Luciferase activities were measured using the Dual Luciferase Assay Kit (Promega) following the manufacturer’s instructions. TGF-β1 promoter was amplified and inserted into a psiCHECK^TM^-2 vector (Promega). 100 ng plasmids and 200 nmol/L NCK1-AS1 mimic or their negative control were transfected into cells (1 x10^5^ per milliliter) using Attractene Transfection Reagent (Qiagen). After transfection for 2 days, the luciferase activity was determined by determining the ratio of firefly to Renilla luciferase activity with a Dual-luciferase reporter system (Promega).

### RT-qPCR

Following RNA isolations using RNAzol reagent, cDNAs were synthesized through reverse transcriptions using SS-IV-RT (Invitrogen). SYBR ® Green Master Mix (Toyobo, Japan) was used to perform qPCRs. The levels of NCK1-AS1 and TGF-β1 mRNA were normalized to 18S rRNA and calculated using the 2^−ΔΔCt^ method [14]. The experiment was repeated 3 times.

### Cell transient transfection

NCK1-AS1 or TGF-β1 expression vector was constructed by Sangon (Shanghai, China). Nucleofector™ Technology was used to achieve transient cell transfections with 10 nM vectors. Cells without transfection (control) and empty vector transfection (negative control) were included to serve as the controls. Subsequent experiments were performed 24 h after transfections. In some experiments, cells were treated with 10 nM TGF-β inhibitor SB431542 (SB, Sigma-Aldrich).

### Transwell assays

EC109 and KYSE150 cells were collected. 3 × 10^3^ cells in 0.1 mL serum-free medium were transferred to the upper chamber, and media with 20% FBS was added into the lower chamber to induce cell movement. Membranes were coated with Matrigel at 37°C for 12 h before invasion assays, while migration assays were carried out using uncoated membranes. At 37°C, cells were cultured for 12 h. Cells on the lower surface of the membranes were stained using 0.5% crystal violet (Sigma-Aldrich) for 15 min in the dark and counted under an optical microscope (Olympus, Japan) [14].

### Western blot

RIPA (Invitrogen) was used to extract total protein from EC109 and KYSE150 cells. After denaturing, proteins were separated on 10% SDS-PAGE gel and transferred onto PVDF membranes. The membranes were blocked at room temperature in 5% nonfat milk for 2 h and incubated subsequently with primary antibodies against GAPDH (ab9485, 1: 1400, Abcam) and TGF-β1 (ab9758, 1:1600, Abcam) and goat anti-rabbit IgG-HRPs secondary antibody (1:1000, MBS435036, MyBioSource). The signals were visualized using ECL (Sigma-Aldrich, USA) and processed using Image J v.1.46 software.

## Statistical analysis

Gene expression levels were expressed by average values of three technical replicates and paired t-test was used for data comparison. ANOVA Tukey’s test was used to compare data of three independent replicates of multiple transfection groups, and data were expressed as mean ± SD. Linear regression was used for correlation analyses. Patients were grouped into low (n = 28) and high (n = 24) plasma NCK1-AS1 level groups based on Youden’s index (cutoff value = 4.17). Survival curves were plotted, and log-rank test was performed for survival curve comparison. Differences with *p*< 0.05 were statistically significant.

## Results

To test our hypothesis that NCK1-AS1 expression is dysregulated in ESCC and this dysregulation carries important clinical significance, we determined its levels in ESCC tissues, paired non-tumor tissues, and plasma of ESCC patients and analyzed their relationship. Moreover, we analyzed and confirmed the binding between NCK1-AS1 and TGF-β1 using the luciferase reporter assay and explored their functions in ESCC by analyzing the effect of their overexpression/inhibition on the migration and invasion of EC109 and KYSE150 cells.

**NCK1-AS1 was upregulated in esophageal squamous cell carcinoma and positively correlated with its plasma level**

**Figure 1.**
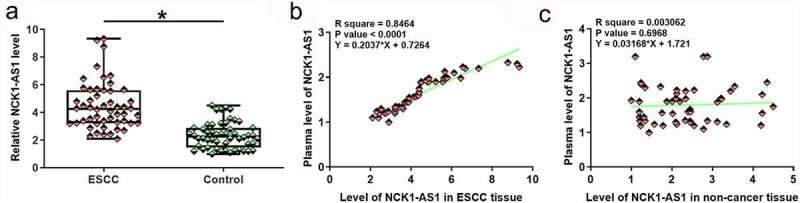
**NCK1-AS1 was upregulated in ESCC tissues and positively correlated with its plasma level**. Expression data analyzed by paired t-test showed that NCK1-AS1 expression was significantly upregulated in ESCC (a). * p < 0.05. Linear regression showed that plasma NCK1-AS1 levels were positively and significantly correlated with its levels in ESCC tissues (b) but not in adjacent non-cancer tissues (c).

NCK1-AS1 expression in ESCC and non-cancer tissues was analyzed by RT-qPCR. Expression data were analyzed by paired t test. It was observed that NCK1-AS1 was significantly upregulated in ESCC tissues compared to non-cancer tissues (Figure 1a, p < 0.05). Plasma levels of NCK1-AS1 were also measured by RT-qPCR. Linear regression was carried out to analyze the correlation between NCK1-AS1 expression in plasma and NCK1-AS1 expression in tissues. NCK1-AS1 expression levels in plasma were positively and significantly correlated with levels of NCK1-AS1 in ESCC tissues (Figure 1b) but not in adjacent non-cancer tissues (Figure 1c).

### High levels of plasma NCK1-AS1 were accompanied by poor survival

**Figure 2.**
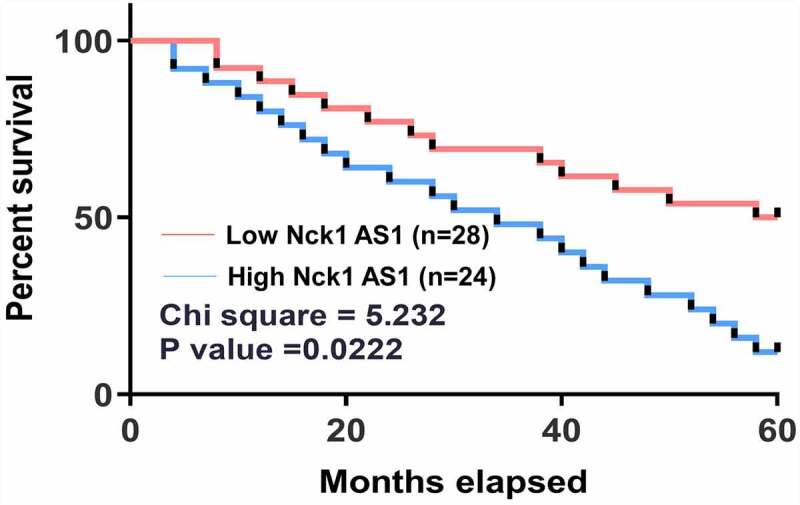
**High plasma NCK1-AS1 levels were correlated with poor survival**. Overall survival condition of patients in the high NCK1-AS1 group was significantly worse than that of patients in the low NCK1-AS1 level group.

No significant differences in levels of plasma NCK1-AS1 were found among different clinical stages. All patients were grouped into high (n = 24) and low (n = 28) plasma NCK1-AS1 level groups based on Youden’s index. Survival curve analysis showed that the overall condition of patients with high NCK1-AS1 levels was significantly worse than that of patients with low NCK1-AS1 levels (Figure 2, Table 1).

### TGF-β1 mRNA was positively correlated with NCK1-AS1 in esophageal squamous cell carcinoma

**Figure 3.**
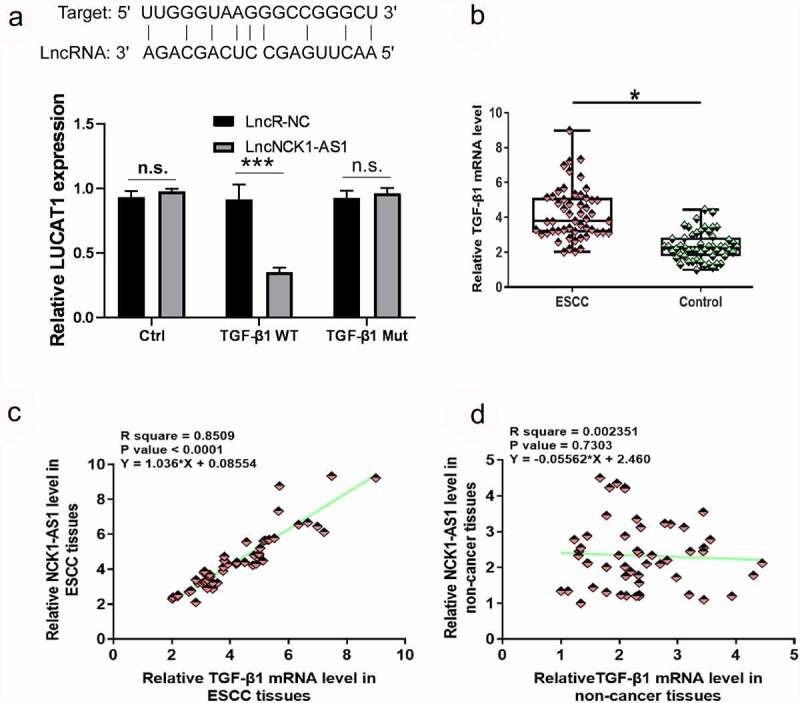
**TGF-β1 mRNA expression was upregulated in ESCC tissues and positively correlated with NCK1-AS1**. Luciferase reporter assay detected that TGF-β1 is related to NCK1-AS1 (a). Expression data analyzed by paired t-test showed that TGF-β1 mRNA expression was significantly upregulated in ESCC tissues compared to non-cancer tissues (b) (* p < 0.05). Linear regression showed that TGF-β1 and NCK1-AS1 were significantly and positively correlated in ESCC tissues (c) but not in adjacent non-cancer tissues (d).

Luciferase reporter assay showed that NCK1-AS1 could interact with TGF-β1 (Figure 3a, p < 0.05) TGF-β1 expression was also analyzed by RT-qPCR. TGF-β1 was significantly overexpressed in ESCC tissues in comparison to non-cancer tissues at mRNA level (Figure 3b, p < 0.05). Correlation analysis showed that TGF-β1 and NCK1-AS1 were significantly and positively correlated across ESCC tissues (Figure 3c) but not in adjacent non-cancer tissues (Figure 3d).

### NCK1-AS1 overexpression stimulated TGF-β1 expression

**Figure 4.**
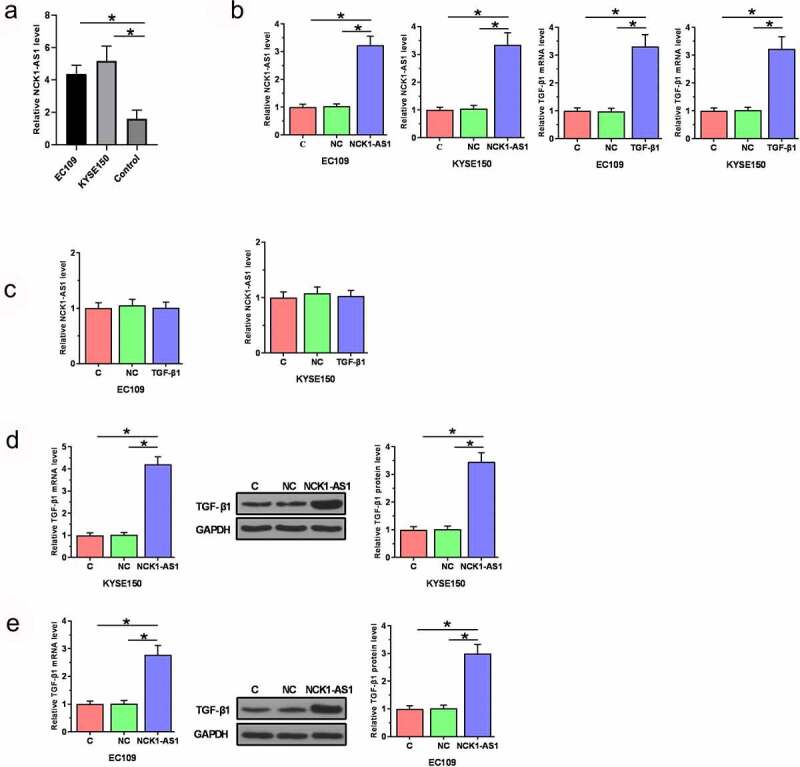
**NCK1-AS1 overexpression stimulated TGF-β1 expression**. Our cells have undergone cell STR identification. Overexpression of TGF-β1 and NCK1-AS1 was confirmed at 24 h after transient transfections (a). TGF-β1 overexpression did not affect NCK1-AS1 (b), while NCK1-AS1 upregulated TGF-β1 expression in ESCC cells (c) (* p < 0.05).

Vectors expressing TGF-β1 and NCK1-AS1 were transfected into EC109 and KYSE150 cells. Overexpression of TGF-β1 and NCK1-AS1 was confirmed at 24 h after transient transfections (Figure 4a, p < 0.05). TGF-β1 overexpression did not significantly affect NCK1-AS1 expression (Figure 4b), while NCK1-AS1 overexpression upregulated TGF-β1 expression in ESCC cells at both mRNA and protein levels (Figure 4c, p < 0.05).

### NCK1-AS1 stimulated esophageal squamous cell carcinoma cell invasion and migration through TGF-β1

**Figure 5.**
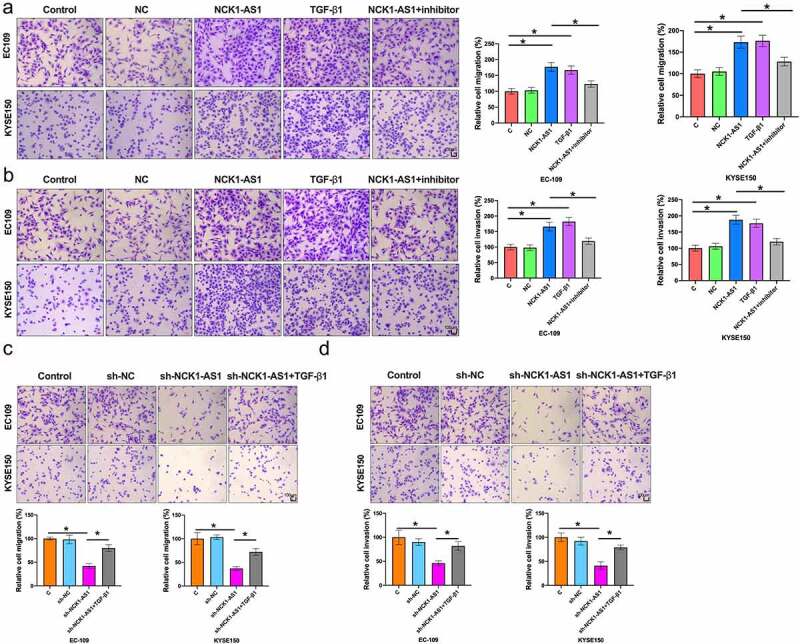
**NCK1-AS1 stimulated ESCC cell migration and invasion through TGF-β1**. TGF-β1 and NCK1-AS1 overexpression increased ESCC cell migration (a) and invasion (b). In addition, TGF-β inhibitor SB431542 attenuated the effect of NCK1-AS1 overexpression. TGF-β1 and sh-NCK1-AS1 affected ESCC cell migration (c) and invasion (d) (* p < 0.05).

Comparing to the two controls (control, C and negative control, NC), TGF-β1 and NCK1-AS1 overexpression increased migration (Figure 5a) and invasion (Figure 5b) rates of ESCC cells (p < 0.05). In addition, incubation with TGF-β inhibitor SB431542 for 24 h reduced the effects of NCK1-AS1 overexpression (p < 0.05). As shown in Figure 5c, NCK1-AS1 silencing prevented migration (Figure 5c) and invasion (Figure 5d) of ESCC cells, while TGF-β1 blocked the function of sh-NCK1-AS1 on ESCC cells. Taken together, NCK1-AS1 stimulated ESCC cell invasion and migration through TGF-β1.

## Discussion

This study highlights NCK1-AS1ʹs function and mechanisms in regulating ESCC metastasis. NCK1-AS1 is an oncogenic lncRNA in cervical cancer [15]. We reported the involvement of NCK1-AS1 in ESCC, explored its prognostic values, and concluded that NCK1-AS1 could upregulate TGF-β1 to promote ESCC.

ESCC is accompanied by the altered expression of a huge number of lncRNAs [16,17]. Some altered lncRNAs participate in ESCC by affecting cancer cell behaviors and chemosensitivity to chemotherapies [16,17]. Our study first showed that NCK1-AS1 was upregulated in ESCC, and NCK1-AS1 overexpression promoted invasion and migration of ESCC cells. Therefore, NCK1-AS1 is also likely an oncogenic lncRNA in ESCC.

LncRNAs are usually expressed during specific developmental or pathological stages to regulate downstream gene expression [18]. However, lncRNAs may enter the blood to traffic systemically, thereby regulating systemic gene expression [19]. We detected NCK1-AS1 in plasma of all ESCC patients. In addition, plasma NCK1-AS1 reflects its expression levels in ESCC tissues. Therefore, we speculated that NCK1-AS1 synthesized in ESCC tissues can be released into the blood, and plasma NCK1-AS1 level can reflect its level in cancer tissue. Comparing the detection of gene expression in tissues, detection of plasma biomarker as a noninvasive approach may be accepted by more patients for disease diagnosis and prognosis. Our study proved that high plasma NCK1-AS1 level was accompanied by poor survival of ESCC patients. Therefore, plasma NCK1-AS1 may serve as a prognostic marker for ESCC. It is worth noting that NCK1-AS1 expression was not significantly affected by clinical stages, which were closely correlated with patients’ survival. Therefore, NCK1-AS1 may be an independent prognostic marker for ESCC.

Our study showed that NCK1-AS1 regulates TGF-β1 as an upstream regulator. This is proven by the following two findings. First, NCK1-AS1 overexpression upregulated TGF-β1 while TGF-β1 overexpression failed to affect NCK1-AS1. Second, TGF-β1 inhibition attenuated the effects of NCK1-AS1 overexpression on cell invasion and migration. TGF-β signaling can be inactivated or activated by certain lncRNAs [20,21]. LncRNAs regulate gene expression mainly at posttranscriptional level, translational level, and epigenetic level [22]. Our study observed that NCK1-AS1 overexpression upregulated TGF-β1 mRNA. Therefore, NCK1-AS1 may affect the stability of TGF-β1 mRNA to regulate TGF-β1 expression. Interestingly, the expression levels of NCK1-AS1 and TGF-β were only closely correlated across ESCC tissue samples but not non-cancer tissue samples. Therefore, the interaction between NCK1-AS1 and TGF-β is likely indirect. However, the factors that mediate the interaction between them remain to be explored.

## Conclusion

NCK1-AS1 expression is up-regulated in ESCC and associated with poor survival outcomes. NCK1-AS1 might affect ESCC cell metastasis by regulating TGF-β1 expression, suggesting that NCK1-AS1 may be a potential prognostic biomarker and novel therapeutic target for ESCC.

## List of abbreviations

esophageal squamous cell carcinoma (ESCC); Long non-coding RNAs (lncRNAs); polyvinylidene fluoride (PVDF).
